# A Block Method Using the Chirp Rate Estimation for NLFM Radar Pulse Reconstruction

**DOI:** 10.3390/s19225015

**Published:** 2019-11-17

**Authors:** Karol Abratkiewicz, Piotr Samczyński

**Affiliations:** Institute of Electronic Systems, Faculty of Electronics and Information Technology, Warsaw University of Technology, 00-665 Warsaw, Poland

**Keywords:** bistatic radar, chirp rate estimation, passive radar, radar countermeasures, radar measurements, electronic warfare, ELINT systems, radar pulse estimation, electromagnetic spectrum-sensing

## Abstract

This paper presents a novel approach to fast and accurate non-linear pulse signal reconstruction dedicated for electromagnetic sensors and their applications such as ELectronic INTelligence (ELINT), electronic warfare (EW), electronic reconnaissance (ER) systems, as well as for passive bistatic radar purposes in which other pulse radars are used as a source of illumination. The method is based on the instantaneous chirp rate (CR) estimation in the time-frequency (TF) domain providing a calculation of the frequency rate between every two consecutive samples. Such a new method allows for the precise reconstruction of the non-linear frequency modulated (NLFM) signal to be carried out in significantly shorter time in comparison to methods known in the literature. The presented approach was tested and validated using both simulated and real-life radar signals proving the usability of the proposed solution in practical applications. The results were compared with the precise extended generalized chirp transform (EGCT) method as a reference technique, using optimal matched filtration as the main concept.

## 1. Introduction

The fast and precise recognition of radar signatures is extremely important on the modern-day battlefield. Effective electronic systems working in real time can give important information to be analyzed by signal intelligence (SIGINT) system operators. This applies to any spectrum-sensing system such as ELINT, ER, SIGINT, EW and jamming systems, for which it is important to obtain precise radar signatures for effective counteraction. This problem is also significant in passive coherent location (PCL) using active radars as illuminators of opportunity, where the technique for radar signal parameter estimation proposed in this paper can also be applied. How important such applications are can be found by analyzing the literature that has been rapidly appearing on this topic in recent years [1,2,3,4,5,6,7,8,9,10,11,12,13]. As described in the aforementioned works, one of the first steps in the signal processing chain of waveform recognition is the estimation of its parameters and their reconstruction. This is because classical pulse radars use different types of frequency modulations. Both linear frequency modulation (LFM) and NLFM with different parameters such as pulse duration, carrier frequency, CR, etc. must be distinguished and estimated. If the used signal is characterized by a linearly modulated frequency, the estimation problem is not complicated, and in the literature many methods can be found. One of the most popular and precise approaches is a fractional Fourier transform [14], which is the generalized Fourier transform allowing intermediate signal representation between the time and frequency domains to be obtained. Cyclostationary analysis is also employed in many applications, but because of noise sensitivity and the requirement of several pulses this approach is not very suitable for radar techniques. Another method uses the maximum-likelihood estimator (MLE) [15], which is accurate but computationally complex. Among the known methods [16,17,18,19,20,21,22] determining LFM signal parameters, an appropriate approach to a given application can be easily found but has significant limitation to LFM signals. However, determining NLFM signal parameters is more complicated and the analysis requires a different methodology. Usually, NLFM radar pulses have a phase described by an even polynomial of degree (e.g., 4, 6, 8 etc.). If the degree of this polynomial is not known a-priori, then the analysis may be difficult. Any implementation using the Wigner or Wigner-Wille transform [23] becomes useless because of the multipath effect occurring in radar signal propagation. In such a case, a direct signal and its copies reflected from the ground provides so-called cross-terms. This disqualifies this approach in the analysis of radar pulses, just as the heterogeneous resolution of a wavelet transform is not an optimal solution for the analysis of radiolocation signals, which are usually relatively wideband. The authors of this article have therefore proposed a novel approach to NLFM signal parameter estimation and reconstruction, which is found further in this article.

This paper is organized as follows: Section 2 contains a short state of the art summary, Section 3 presents the theory of CR estimation in the time-frequency domain. Section 4 covers the proposed method, while Section 5 and Section 6 present the results obtained for the proposed approach for simulated and real-life signals. The article closes with a summary and discussion.

## 2. State of the Art

Due to the non-stationary nature of radar pulsed signals, such as NLFM pulses for example, the TF analysis is often applied. Such signals are commonly used in radar systems because of the reduced side-lobe level of the waveform filtered using a matched filter [24,25,26]. In the literature, the problem of signal reconstruction is widely described. The synthesis methods, as well as the estimation of NLFM signal parameters, are developed and discussed. The issue is known both in radars [27,28,29] and sonars [30,31]. Unfortunately, most of these methods require certain assumptions, such as the phase being described by the *P*-th order polynomial. Using a short-time Fourier transform (STFT) Djurović and Stanković proposed in [32] an instantaneous frequency (IF) estimator, providing an obtainable, accurate phase reconstruction. A similar solution, described by the same authors, dealing with the maximum-likelihood estimator was presented in [33]. In [34] the adaptive short-time Fourier transform (STFT) was used to estimate IF, which makes it a comparable approach to the one mentioned above. A very precise approach is presented in [35], where the signal described by the polynomial phase is processed using a discrete polynomial transformation. Also, in this case, it is not possible to use this method due to the assumption of constant signal amplitude. In radar systems, the analog frontend usually has individual characteristics (the gain and matching properties of microwave components) that are not constant for the entire device’s operating band. In this case, the modulation causes a non-linearity of amplitude which can be significant. Therefore, it is not possible to use this method in this solution. In general, there are other algorithms for estimating the phase described by a polynomial, examples of which can be found in [36,37,38].

The most accurate method for the estimation of radar pulse signal parameters is the extended generalized chirp transform (EGCT), assuming that the phase of the pulse is described by the *P*-th order polynomial (typically *P* is an odd number) [39,40,41]. In fact, this method is based on the maximum-likelihood estimation process, which is slower than other known techniques, but very accurate. In this case, the signal and its phase are expressed by the following equations (respectively Equations (1) and (2)):

(1) $$y\left(t\right)=A_{y}\cdot \exp\left(j\Phi_{y}\left(t\right)\right),$$

(2) $$\Phi_{y}\left(t\right)=\sum_{i=0}^{P}a_{i}t^{i},$$

where $A_{y}\in R$ is the amplitude of the signal and $\Phi_{y}\left(t\right)$ is its phase described by the *P*-th order polynomial with coefficients $a_{i}$. The EGCT algorithm uses matched filter principles determined by the equation:

(3) $$D\left(A\right)=\int_{t_{1}}^{t_{2}}y\left(t\right)\cdot \exp\left(-jP_{y}\left(A\right)\right)dt,$$

for the given vector of the coefficients $A=[a_{0},a_{1},\ldots ,a_{P}]$ and a phase expressed by the same polynomial as $\Phi_{y}$:

(4) $$P_{y}\left(A\right)=\sum_{i=0}^{P}a_{i}t^{i}.$$

In the next step, a MLE is used for the *A* coefficients, finding the maximum EGCT value:

(5) $$\tilde{A}=\arg\left\lfloor \max_{A}\left|D(A)\right|\right\rfloor ,$$

where ⌊·⌋ expresses floor function, whereas |·| denotes the absolute value. Based on the matched filtration, subsequent phase coefficients from the reference signal are estimated. The disadvantage of this method is the computational complexity as well as the knowledge of the *P* polynomial degree being required. Otherwise, calculations should be performed for all values from a given range. However, the method is ineffective when the pulse used does not have the phase described by the polynomial. Then the reconstruction of the signal can be inaccurate. This article presents a new approach to signal reconstruction intended for use in ELINT systems and/or in passive radar technology using other radars as a source of illumination, in which the reference signal after the parameter estimation is presented as a waveform with a variable frequency rate. The following research is based on previous experiments confirming the applicability of the CR estimation approach in radiolocation systems [41]. Due to positive results being obtained, a novel, extended version of the already described method was developed, which allows not only the estimation of the linear component in the radar pulse, but also non-linear components.

## 3. Chirp Rate Estimation in the Time-Frequency Domain

The concept of using a complex phase of the STFT for the CR estimation of non-stationary signals in the TF domain is described in [42]. According to this method, the multi component signal is denoted in the following manner:

(6) $$x\left(t\right)=\sum_{n=1}^{N}u_{n}\left(t\right)=\sum_{n=1}^{N}a_{n}\left(t\right)\exp\left(j\Phi_{n}\left(t\right)\right),$$

where x(t) is the considered signal, $a_{n}\left(t\right)$ denotes its envelope, whereas the instantaneous phase of the *n*-th order component is expressed as $\Phi_{n}\left(t\right)$. However, if the considered signal contains only one dominant component, Equation (6) can be simplified and rewritten as:

(7) $$x\left(t\right)=A_{x}\exp\left(j\Phi_{x}\left(t\right)\right),$$

characterized by the amplitude $A_{x}$∈R and phase described as:

(8) $$\Phi_{x}\left(t\right)=\phi_{x}+\omega_{x}t+2\pi \cdot \alpha t^{2}/2=\phi_{x}+2\pi t\left(f_{0}+\alpha t/2\right),$$

where $\omega_{x}=2\pi f_{0}$ is the angular frequency with the carrier frequency $f_{0}$, and α is the CR. For such a signal, STFT can be calculated using an analyzing window h(t), which leads to TF distribution expressed as follows [43]:

(9) $$F_{x}^{h}\left(t,\omega \right)=\int_{R}x\left(\tau \right)h\left(t-\tau \right)e^{-j\omega \tau }d\tau =A_{x}^{h}\left(t,\omega \right)e^{j\phi_{x}^{h}\left(t,\omega \right)}=e^{\Lambda_{x}^{h}\left(t,\omega \right)+j\phi_{x}^{h}\left(t,\omega \right)},$$

where STFT phase is defined as $\phi_{x}^{h}\left(t,\omega \right)$, $A_{x}^{h}\left(t,\omega \right)$ denotes STFT absolute value ($A_{x}^{h}\left(t,\omega \right)>0$) and $\Lambda_{x}^{h}\left(t,\omega \right)=\ln\left(A_{x}^{h}\left(t,\omega \right)\right)$. For the frequency modulated (FM) signal, the instantaneous CR can be described by the K estimator:

(10) $$K\left(t,\omega \right)=-\left(\frac{\partial \Lambda_{x}^{h}\left(t,\omega \right)}{\partial t}/\frac{\partial \Lambda_{x}^{h}\left(t,\omega \right)}{\partial \omega }\right),$$

and can be extended for amplitude modulated (AM) FM signals as described in [42]. Auger et al. in [44] showed that using the concept of complex phase of the STFT the expressions $\partial F_{x}^{h}\left(t,\omega \right)/\partial t$ and $\partial F_{x}^{h}\left(t,\omega \right)/\partial \omega$ can be replaced by using a modified analysis window. Using Equation (9), it can be assumed that:

(11) $$\partial F_{x}^{h}\left(t,\omega \right)/\partial t=\int x\left(\tau \right)\frac{\partial h\left(t-\tau \right)e^{j\omega (t-\tau )}}{\partial t}d\tau =F_{x}^{Dh}\left(t,\omega \right),$$

as well as:

(12) $$\partial F_{x}^{h}\left(t,\omega \right)/\partial \omega =\int x\left(t-\tau \right)h\left(\tau \right)\frac{\partial e^{j\omega \tau }}{\partial \omega }d\tau =\int x\left(t-\tau \right)h\left(\tau \right)j\tau e^{j\omega \tau }d\tau =jF_{x}^{Th}\left(t,\omega \right),$$

which leads to:

(13) $$\frac{\partial \ln(F_{x}^{h}\left(t,\omega \right))}{\partial t}=\frac{\partial F_{x}^{h}\left(t,\omega \right)}{\partial t}\frac{1}{F_{x}^{h}\left(t,\omega \right)}=\frac{F_{x}^{Dh}\left(t,\omega \right)}{F_{x}^{h}\left(t,\omega \right)}$$

and

(14) $$\frac{\partial \ln(F_{x}^{h}\left(t,\omega \right))}{\partial \omega }=\frac{\partial F_{x}^{h}\left(t,\omega \right)}{\partial \omega }\frac{1}{F_{x}^{h}\left(t,\omega \right)}=j\frac{F_{x}^{Th}\left(t,\omega \right)}{F_{x}^{h}\left(t,\omega \right)}$$

Equation (11) describes STFT in which the analysis window h(t) is differentiated, while Equation (12) describes STFT where the window is multiplied by the time ramp. Considering Equations (11) and (12) and calculating the higher derivatives of the complex phase, three CR estimators in the TF domain are to be determined as follows [45]:

(15) $$K\left(t,\omega \right)=-\left(\frac{\partial \Lambda_{x}^{h}\left(t,\omega \right)}{\partial t}/\frac{\partial \Lambda_{x}^{h}\left(t,\omega \right)}{\partial \omega }\right)=\frac{R\left\{\frac{F_{x}^{Dh}\left(t,\omega \right)}{F_{x}^{h}\left(t,\omega \right)}\right\}}{ℑ\left\{\frac{F_{x}^{Th}\left(t,\omega \right)}{F_{x}^{h}\left(t,\omega \right)}\right\}},$$

(16) $$D\left(t,\omega \right)=-\left(\frac{\partial^{2}\Lambda_{h}\left(t,\omega \right)}{\partial t^{2}}/\frac{\partial^{2}\Lambda_{h}\left(t,\omega \right)}{\partial \omega \partial t}\right)=\frac{R\left\{\frac{F_{x}^{D^{2}h}\left(t,\omega \right)}{F_{x}^{h}\left(t,\omega \right)}-{\left[\frac{F_{x}^{Dh}\left(t,\omega \right)}{F_{x}^{h}\left(t,\omega \right)}\right]}^{2}\right\}}{ℑ\left\{\frac{F_{x}^{\mathrm{DT}h}\left(t,\omega \right)}{F_{x}^{h}\left(t,\omega \right)}-\frac{F_{x}^{Dh}\left(t,\omega \right)}{F_{x}^{h}\left(t,\omega \right)}\frac{F_{x}^{Th}\left(t,\omega \right)}{F_{x}^{h}\left(t,\omega \right)}\right\}},$$

(17) $$F\left(t,\omega \right)=-\left(\frac{\partial^{2}\Lambda_{h}\left(t,\omega \right)}{\partial \omega \partial t}/\frac{\partial^{2}\Lambda_{h}\left(t,\omega \right)}{\partial \omega^{2}}\right)=-\frac{ℑ\left\{\frac{F_{x}^{\mathrm{DT}h}\left(t,\omega \right)}{F_{x}^{h}\left(t,\omega \right)}-\frac{F_{x}^{Dh}\left(t,\omega \right)}{F_{x}^{h}\left(t,\omega \right)}\frac{F_{x}^{Th}\left(t,\omega \right)}{F_{x}^{h}\left(t,\omega \right)}\right\}}{R\left\{\frac{F_{x}^{T^{2}h}\left(t,\omega \right)}{F_{x}^{h}\left(t,\omega \right)}-{\left[\frac{F_{x}^{Th}\left(t,\omega \right)}{F_{x}^{h}\left(t,\omega \right)}\right]}^{2}\right\}}.$$

In Equations (15)–(17) ℜ is the real part and ℑ is the imaginary part. There are some fundamental differences between the estimators [41,45]:

1. The K estimator is distorted by an uncertainty occurring in the middle of the energy ridge, which does not appear in the D and F estimators. This was the reason higher order derivatives of the complex phase were proposed.
2. The K estimator assumes constant amplitude.
3. The D and F estimators are characterized by a higher number of mathematical operations.
4. The last major difference relates to the statistical properties of the estimators and the resistance of noise.

**Figure 1:**
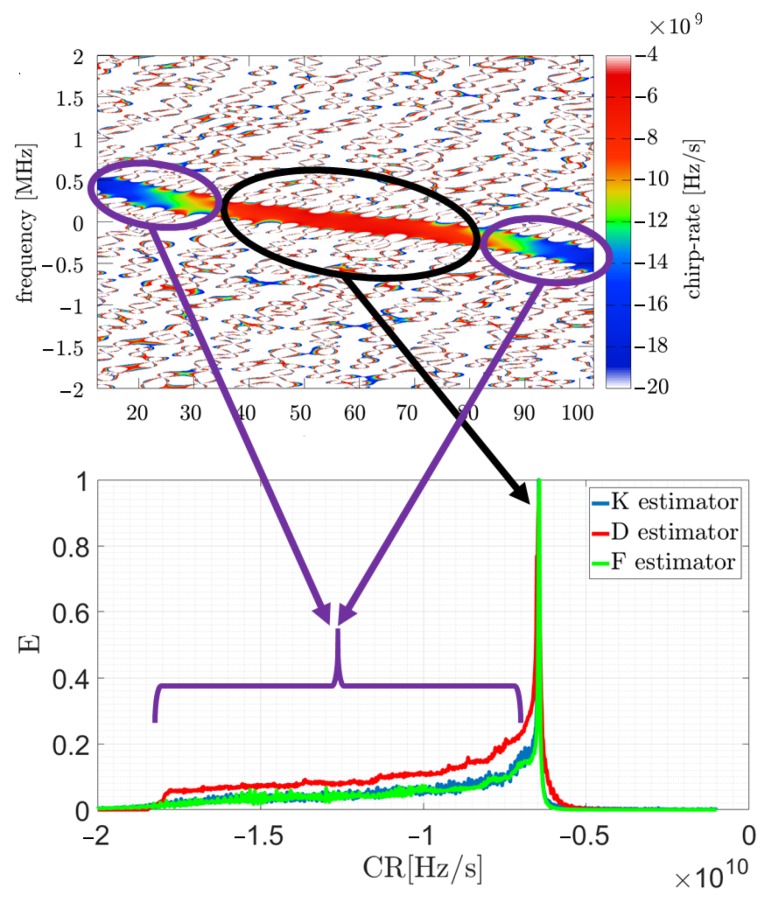
Accelerogram (**above**) and R profile (**below**) of the simulated NLFM radar pulse with the corresponding parts marked. For a more detailed description see [41], *©* 2019 IEEE.

In addition, the statistical effectiveness of the three estimators was presented by the authors in [46], where the variance for each of the tools used was compared with the Cramer-Rao lower bound. The CR estimation approach was effectively used to analyze acoustic signals [47], sonar [48] and radar NLFM pulses [41]. The last of the mentioned papers in particular shows that the usage of the K, D and F estimators is an effective solution in the analysis of signals from pulse radars. The NLFM signal (simulated and received from the Air Traffic Control (ATC) radar) was presented there in the form of an accelerogram and R profile (calculated for overall TF plane), which is a histogram, assigning energy (E) to a given CR value from an assumed range. This approach was proposed in [42] and used in [41,47,48] as an efficient tool to assess the CR of dominant components. An exemplary phase accelerogram and normalized R profile are shown in Figure 1.

The largest part of the energy is concentrated around the linear frequency modulated signal part. Other, non-linear parts of the signal appear on the R profile with a set of values marked in purple. Such an approach provides general information about the energy distribution in the function of CR, resulting in the neglecting of time dependencies. This prompted the authors to analyze NLFM signals and extract the instantaneous CR for subsequent moments associated with the highest energy. The main idea was to verify whether it is possible to determine the dominant CR not for the entire plane, but for consecutive time cuts in the TF plane. The considered approach would provide an estimation of a 3D R profile and based on this distribution, further reconstruction would be carried out, which is described in detail in the next sections.

## 4. Description of the Proposed Method

The proposed BRM (Block Reconstruction Method) algorithm is based on the properties of the estimators described by Equations (15)–(17), which consist of determining the instantaneous frequency rate of the signal in each point on the TF plane. One of the STFT interpretations describing the TF distribution of a signal’s energy is a part of the waveform multiplied by the analysis window h(t), and a discrete Fourier transform is performed on the result of this operation. Next, the window slides along the time axis, resulting in a 2D signal representation. Subsequent analyzed parts of the signal may overlap, and the number of samples by which the window moves in each iteration is called hop-size. In the most accurate case, the hop-size in the STFT operation is equal to one (H=1). Based on the distribution provided in this way, an accelerogram is calculated showing the instantaneous CR on the TF plane. One of the three estimators presented can be used for this purpose; however, as was shown in [41], the F estimator is characterized by the best accuracy in the presence of noise and it will be used in further considerations.

**Figure 2:**
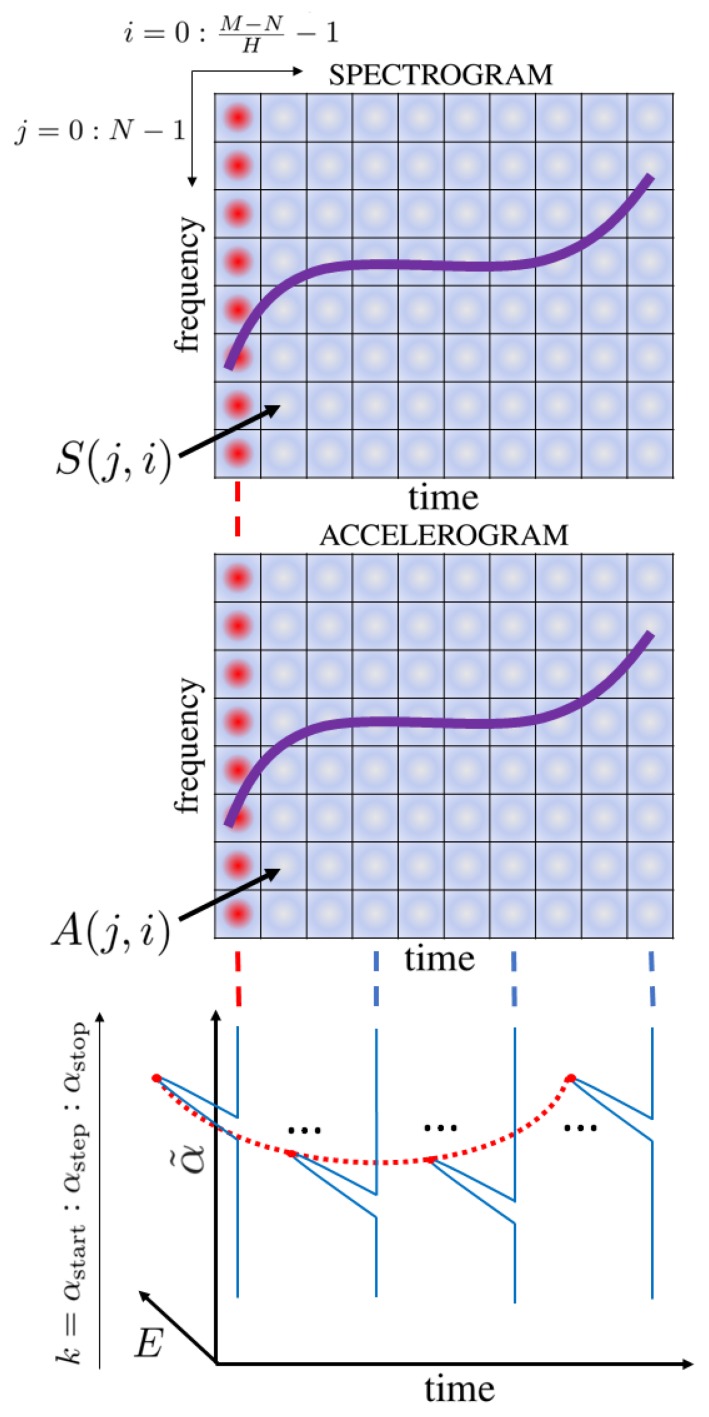
3D R profile calculation scheme. In the above diagram: *M*—signal length, *N*—FFT size, *H*—hop-size, *S*—spectrogram, *A*—accelerogram, $\tilde{\alpha }$—estimated CR, *E*—energy.

Based on the obtained energy and CR distributions, it is possible to calculate the R profile mentioned in the previous section presenting energy associated with the CR. The disadvantage mentioned in the previous section related to the lack of information as to how the signal energy assigned to a particular CR changes in time has led the authors to take a different approach presented in this paper. Herein, the authors proposed an extended processing method consisting of analyzing the modified R profile; however, it is not calculated for the entire plane but for the consecutive moments. This corresponds to the columns in the 2D TF distribution. The general idea is shown in Figure 2 and described by a pseudo-code in Algorithm 1.
**Algorithm 1:** 3D R profile calculation algorithm.
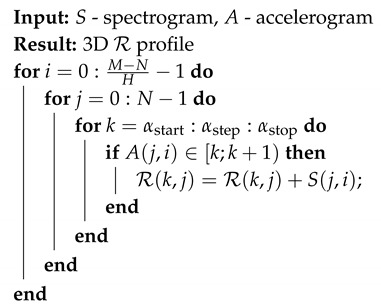


**Figure 3:**
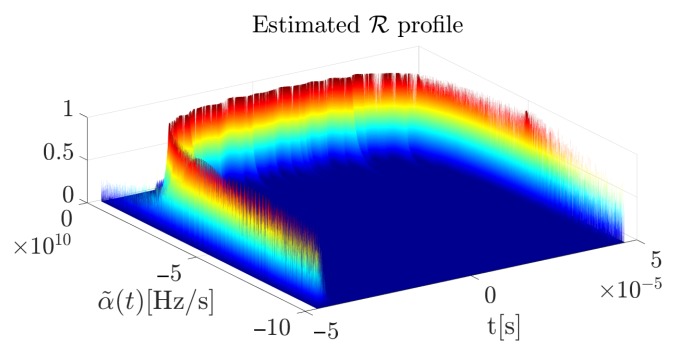
Estimated 3D R profile obtained for the exemplary NLFM pulse.

The interpretation of such a 3D R profile is an expression of how much energy of the considered signal is related to a specific CR from a given range for a particular time sample. In comparison to the R presented in [41], the 3D R profile treats the signal in a more detailed way, without generating CR-energy relationships to a single graph but a 3D relationship time-CR-energy. This is particularly significant in the analysis of NLFM signals, because the usual R profile can extract only the dominant component, which leads to consideration of LFM signals. In the case of an NLFM waveform this assumption is not valid, so the authors have created a solution that allows more robust and rigorous analysis to be carried out. Through the modification of a known approach, additional signal information is delivered. Furthermore, there are no dependencies between the type of analyzed signal (polynomial phase, linear or non-linear frequency modulation, etc.) and the estimated CR, which changes from sample to sample. By analyzing the R profile for each column, a non-linear component with significant energy (but smaller than the dominant component in the whole plane) can be extracted, which is especially important in the analysis of NLFM radar pulses. In such a case, the maximum value of the R profile in each column provides information about the instantaneous CR in the signal under consideration, and thus direct information about the signal properties. What is important, this method does not require initial information about the signal parameters as it is present in the accurate but computationally complex EGCT method. According to the proposed method the 3D R profile was calculated for an exemplary NLFM signal which is presented in Figure 3. The useful part of the distribution is created by the maximum value of $\tilde{\alpha }$ for each particular profile along the time axis (red dotted curve).

The estimated CR curve can be characterized by the following expression:

(18) $$\tilde{\alpha }\left(t\right)=\underset{k}{arg max}\left(R\left(k,t\right)\right),$$

where $\tilde{\alpha }\left(t\right)$ is the estimated CR curve, R is the 3D profile and *k* is the set of the considered CR values (see Figure 2 and Algorithm 1). The introduced curve allows the reconstructed signal to be expressed in the following manner (in general in the continuous time domain):

(19) $$\tilde{x}\left(t\right)=\exp\left(j2\pi \left(\frac{\tilde{\alpha }\left(t\right)t^{2}}{2}\right)\right).$$

As was mentioned in Section 3, a typical NLFM radar pulse is characterized by a reduced side-lobe level of the signal at the output of the matched filter. This allows smaller targets which are characterized by a lower radar cross section (RCS) to be detected when in close range of a strong target with a higher RCS. Use of this type of waveform is similar to multiplying a signal by a window function which provides a reduced side-lobe level and a wider main lobe. Such a waveform is described by Equation (1) with the phase defined by the *P*-th order polynomial. In general, it can be any order, but in practical applications only the first few phase components are usually considered. In this paper, the polynomial is reduced to P=10 which leads to the expression:

(20) $$\Phi_{x}\left(t\right)=a_{0}+a_{1}t+a_{2}t^{2}+a_{4}t^{4}+a_{6}t^{6}+a_{8}t^{8}+a_{10}t^{10}.$$

In real-life systems, usually four of them appear simultaneously: $a_{0}$—initial phase, $a_{1}$—carrier frequency, $a_{2}$—CR, and $a_{4}$/$a_{6}$/$a_{8}$/$a_{10}$—non-linear component (usually only one of them). Because the phase models described by Equations (8) and (20) are different, it is necessary to modify the second one to the appropriate form. To obtain this, Equation (20) will be normalized by a 2π factor and differentiated with respect to time. It will also be assumed that the initial phase $a_{0}=0$ and the signal are analyzed in the base band, which leads to $a_{1}=0$. This provides the following expression describing the typical signal models:

(21) $$x_{4}\left(t\right)=A_{x}\exp\left(j2\pi \left(\frac{\alpha t^{2}}{2}+\frac{\beta t^{4}}{4}\right)\right),$$

(22) $$x_{6}\left(t\right)=A_{x}\exp\left(j2\pi \left(\frac{\alpha t^{2}}{2}+\frac{\gamma t^{6}}{6}\right)\right),$$

(23) $$x_{8}\left(t\right)=A_{x}\exp\left(j2\pi \left(\frac{\alpha t^{2}}{2}+\frac{\delta t^{8}}{8}\right)\right),$$

(24) $$x_{10}\left(t\right)=A_{x}\exp\left(j2\pi \left(\frac{\alpha t^{2}}{2}+\frac{\varepsilon t^{10}}{10}\right)\right),$$

where the lower index *P* in the $x_{P}\left(t\right)$ expression describes the polynomial order, $\alpha =\frac{a_{2}}{\pi }$, $\beta =\frac{2a_{4}}{\pi }$, $\gamma =\frac{3a_{6}}{\pi }$, $\delta =\frac{4a_{8}}{\pi }$ and $\varepsilon =\frac{5a_{10}}{\pi }$. In general, the CR estimation approach consists of calculating the second order derivative of the phase with respect to time. If any higher order components occur, they are also treated as α. By calculating the second order derivative of the phase of the signals described by Equations (21)–(24), the coefficients corresponding to the degree of the polynomial describing the signal will be determined as follows:

(25) $$\frac{d^{2}\Phi_{4}\left(t\right)}{dt^{2}}=2\pi \left(\alpha +3\beta t^{2}\right),$$

(26) $$\frac{d^{2}\Phi_{6}\left(t\right)}{dt^{2}}=2\pi \left(\alpha +5\gamma t^{4}\right),$$

(27) $$\frac{d^{2}\Phi_{8}\left(t\right)}{dt^{2}}=2\pi \left(\alpha +7\delta t^{6}\right),$$

(28) $$\frac{d^{2}\Phi_{10}\left(t\right)}{dt^{2}}=2\pi \left(\alpha +9\varepsilon t^{8}\right).$$

The α component is given directly as a result of the CR estimation process, whereas additional components, considering the presented factors, provide supplementary information about phase changes. It is necessary to remember at this point that each of the estimators provides the result divided by 2π disabling this factor from Equations (25)–(28). In the case when an illuminating radar transmits a signal with unknown parameters, their estimation can be complex and inaccurate, especially when the polynomial order is not known. In such a situation, non-linear components are considered to be an instantaneous CR. In the next section, it is shown that the proposed method can reconstruct a signal even if the initial model of the signal is unknown in the reference receiver.

## 5. Results for the Synthetic Signal

To verify the algorithm, a pulse signal was analyzed. The waveform is given by the following equation:

(29) $$x\left(t\right)=\exp\left(j\Phi_{x}\left(t\right)\right),$$

where $\Phi_{x}\left(t\right)$ is the phase of the signal defined as the 6-th order polynomial function (with coefficients $a_{2}$ and $a_{6}$) expressed as:

(30) $$\Phi_{x}\left(t\right)=a_{2}t^{2}+a_{6}t^{6},$$

which gives a waveform described by the following model:

(31) $$x\left(t\right)=\exp\left(j(-1\cdot 10^{10}t^{2}-3\cdot 10^{27}t^{6})\right).$$

**Figure 4:**
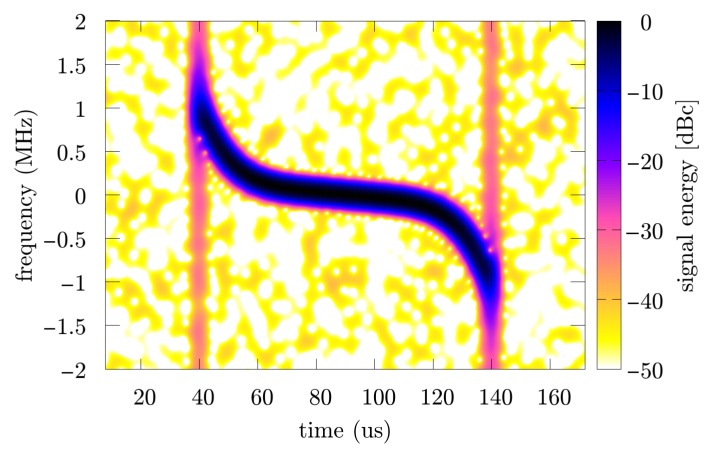
Spectrogram of the simulated pulse.

**Figure 5:**
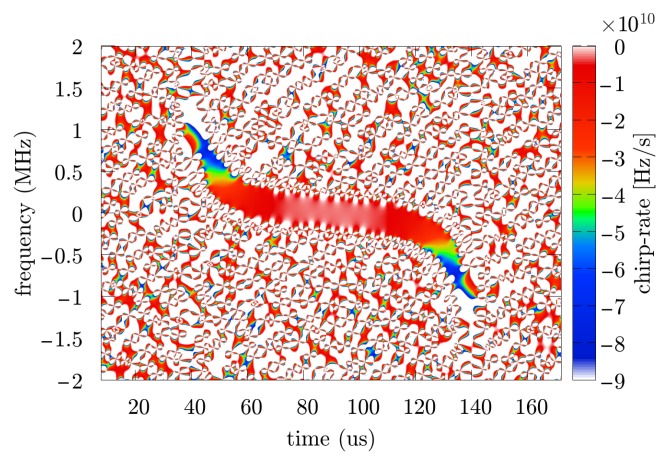
Accelerogram of the simulated pulse.

Further research was carried out based on three SNR levels (10, 20 and 30 dB), and for each value from this range 250 times of the Monte Carlo simulation were prepared with individual noise realization. In the considered case, when the illuminator of opportunity is another pulse radar, the transmitted signal is usually characterized by a high power of several dozen kW or more. In addition, a directional antenna with low-noise amplifiers and a set of band-pass filters is used to receive such a signal. In this case, it seems to be sufficient to assume that the SNR is greater than or equal to 10 dB. An exemplary spectrogram and accelerogram of the considered signal are presented respectively in Figure 4 and Figure 5.

For all data sets $\tilde{\alpha }\left(t\right)$ was estimated as a curve related to the maximum value of the 3D R profile introduced in the previous section, and in order to uniform each curve, polynomial approximation was carried out.

The matched filtration procedure is expressed as:

(32) $$y\left(\tau \right)=\int_{-\infty }^{\infty }x{\left(\tau -t\right)}^{*}g\left(t\right)dt,$$

where g(t) is the matched filter impulse response given by the formula:

(33) $$g\left(t\right)=x^{*}\left(t-\tau \right),$$

and is directly related to the cross-correlation function given by the equation:

(34) $$\chi \left(\tau \right)[1pt]\overset{\Delta }{=}\int_{-\infty }^{\infty }x_{r}{\left(t\right)}^{*}x_{s}\left(t+\tau \right)dt,$$

where $x_{r}$ is the reference (reconstructed) signal and $x_{s}$ is the surveillance (received) signal. From the application point of view, cross-correlation analysis is enough to compare the reconstructed signal and describe its parameters as an illuminating waveform in radar techniques.

**Figure 6:**
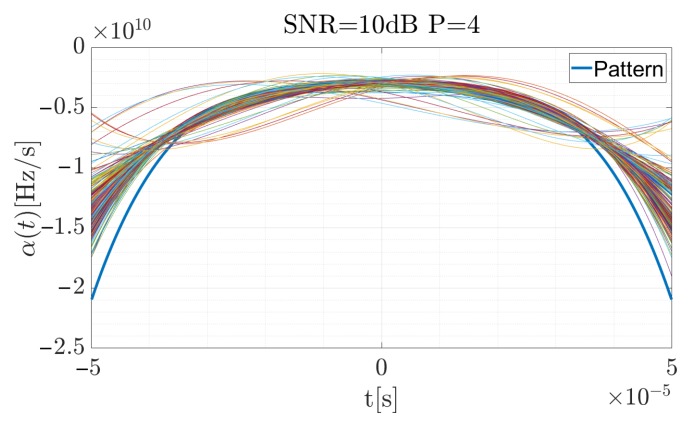
Approximated $\tilde{\alpha }\left(t\right)$ for the simulated pulse with SNR = 10 dB for 250 times of the Monte Carlo simulation.

**Figure 7:**
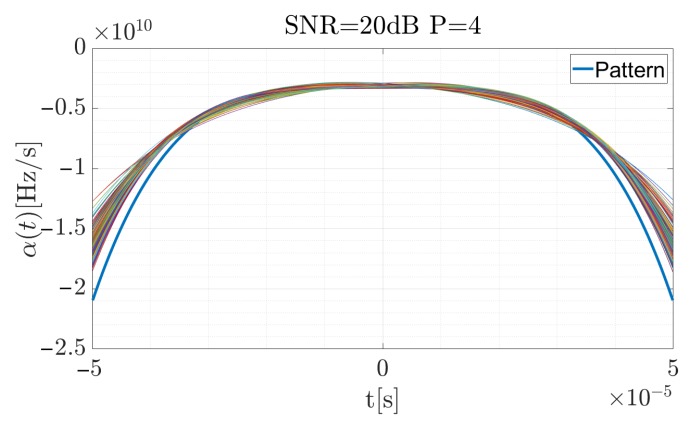
Approximated $\tilde{\alpha }\left(t\right)$ for the simulated pulse with SNR = 20 dB for 250 times of the Monte Carlo simulation.

**Figure 8:**
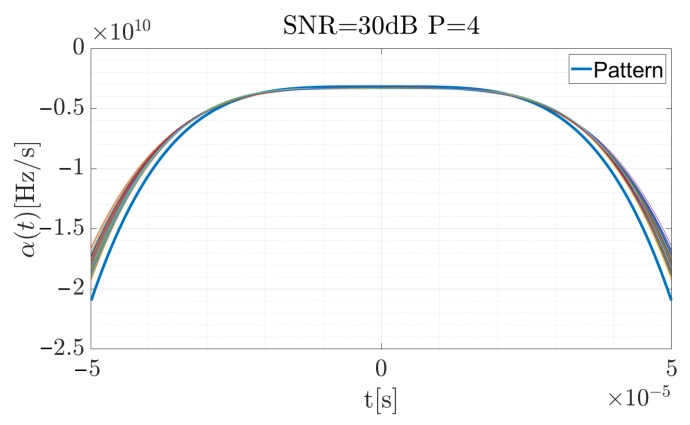
Approximated $\tilde{\alpha }\left(t\right)$ for the simulated pulse with SNR = 30 dB for 250 times of the Monte Carlo simulation.

Figure 6, Figure 7 and Figure 8 present the reference CR curve (in blue) and 250 times of the Monte Carlo simulation estimated using Equation (17) and approximated by the P=4 order polynomial functions for different SNR values. The TF distribution was obtained using a Blackman-Harris window, with the window width equal to 500 samples while the sampling rate was $f_{s}=5$ MHz.

**Figure 9:**
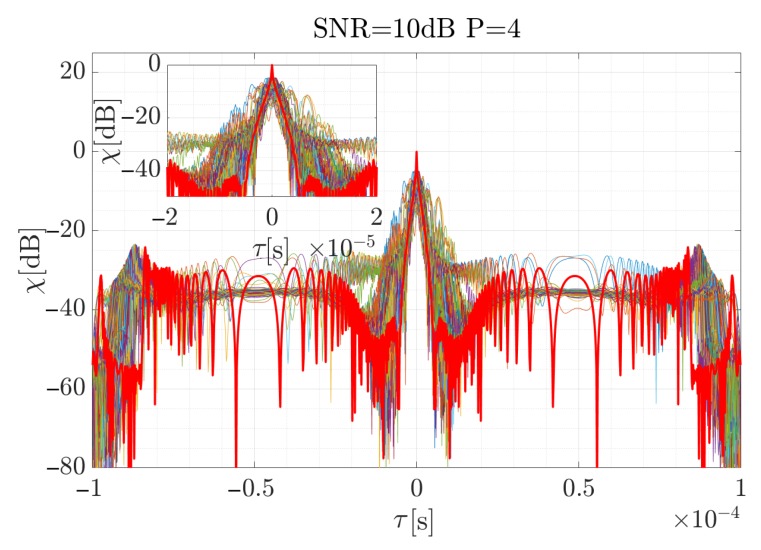
Normalized cross-correlation function for the reconstructed simulated signal when SNR = 10 dB for 250 times of the Monte Carlo simulation. Red curve depicts reference obtained for the EGCT algorithm.

**Figure 10:**
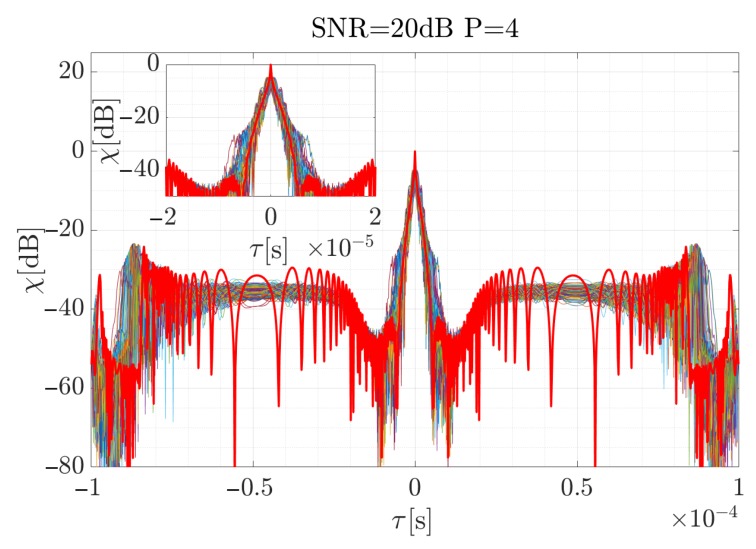
Normalized cross-correlation function for the reconstructed simulated signal when SNR = 20 dB for 250 times of the Monte Carlo simulation iterations. Red curve depicts reference obtained for the EGCT algorithm.

**Figure 11:**
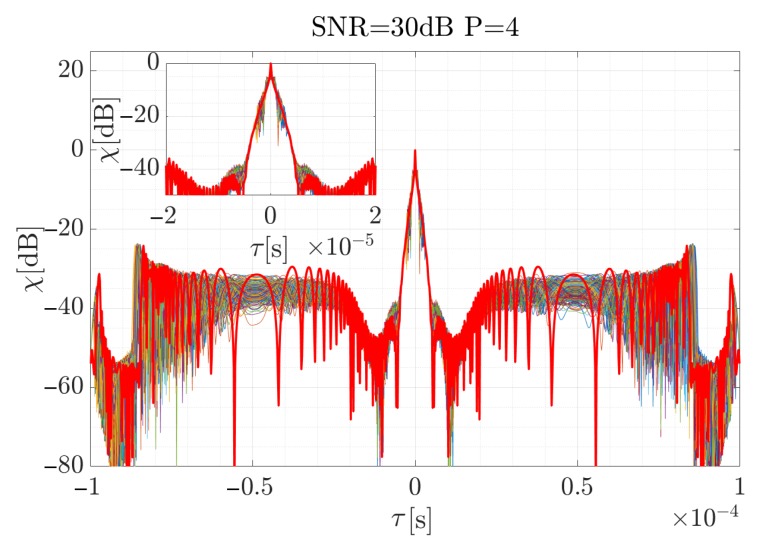
Normalized cross-correlation function for the reconstructed simulated signal when SNR = 30 dB for 250 times of the Monte Carlo simulation iterations. Red curve depicts reference obtained for the EGCT algorithm.

For the high SNR values the estimation is accurate, and the highest differences occur at the beginning and the end of the curve, when the CR between the consecutive steps rapidly changes. According to Equation (19), the signal was reconstructed (considering the factors introduced in the previous section for the signal described by the 4-th order polynomial) and using Equation (34), the cross-correlation function for each case was computed. The results are presented in Figure 9, Figure 10 and Figure 11. The red curve is the reference provided by the EGCT analysis. The benchmark EGCT approach is widely described in the literature, so the particular steps will not be presented in this paper.

As is shown, the EGCT analysis, which is the most accurate but computationally complex, provides comparable results of the signal reconstruction. Both the width of the main lobe and the side-lobe level are similar which confirms the usability of the proposed method. In the next section a real-life signal is analyzed using the introduced approach.

## 6. Results for the Real-Life Radar Signal

A real-life signal was examined to verify the BRM approach. The waveform comes from an air traffic control (ATC) radar that ensures the safety of the airspace around Warsaw’s Chopin Airport. The data collection was done using an NI PXI vector signal analyzer [49]. The main parameters of the radar are as follows:

- carrier frequency: $f_{c_{1}}=2.8$, $f_{c_{2}}=2.801$, $f_{c_{3}}=2.83$, $f_{c_{4}}=2.831$ [GHz],
- pulse repetition frequency: $f_{\mathrm{PRF}}=825$ [Hz],
- pulse duration: $T_{D_{1}}=1$, $T_{D_{2}}=100$ [μs],
- transmitted power: $P_{T}=19.5$ [kW],
- antenna gain: $G_{A}=34$ [dB].

**Figure 12:**
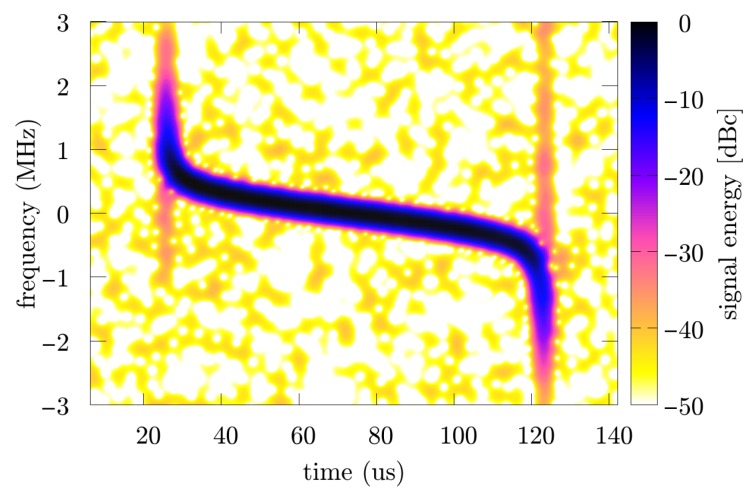
Spectrogram of the gathered ATC radar pulse.

**Figure 13:**
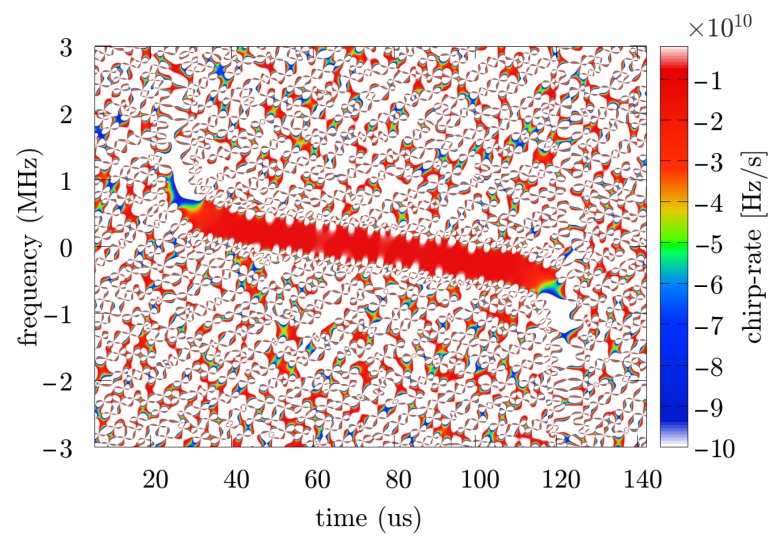
Accelerogram of the gathered ATC radar pulse.

The part containing several pulses was recorded, then a single pulse was selected, and the phase parameters were calculated using the two methods discussed—the proposed approach and the reference method. The sampling rate of the signal was $f_{s}=40$ MHz. An exemplary spectrogram and accelerogram are presented respectively in Figure 12 and Figure 13.

**Figure 14:**
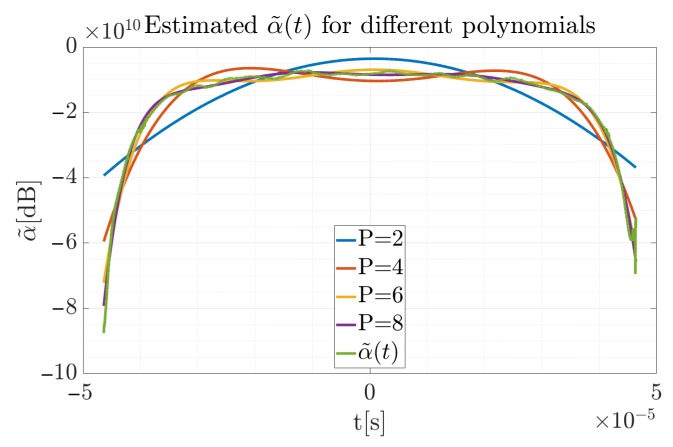
Estimated (green) and approximated $\tilde{\alpha }\left(t\right)$ curves for different degree of the polynomial for the recorded signal.

In contrast to the simulated pulse, the true degree of the polynomial is not known in this case, therefore it is necessary to verify for which degree the approximation error is the smallest. Taking into account the introduced factors, for each polynomial order $\tilde{\alpha }\left(t\right)$ was estimated, which is presented in Figure 14.

**Figure 15:**
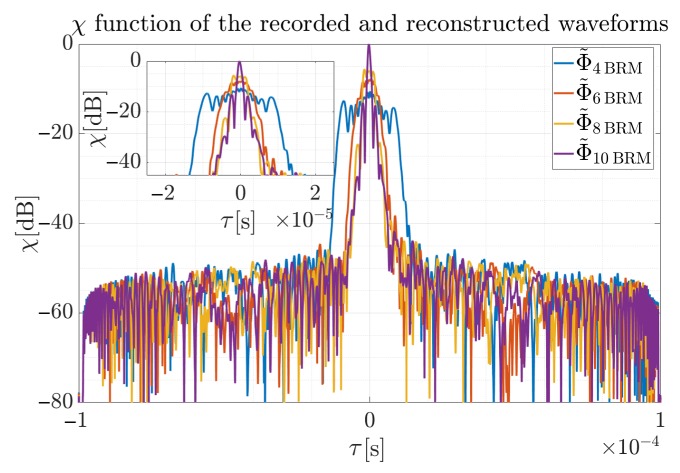
Normalized cross-correlation function of the recorded signal and reconstructed waveform for the analyzed polynomial orders. Reconstructed signal assumed different phase polynomial orders and was provided by the proposed method.

As can be noted, the most accurate approximation was obtained for P=8. Additionally, the cross-correlation function was employed to allow maximum peak values for different polynomials’ reconstructed signals to be compared. Results for the χ function for four considered orders are presented in Figure 15.

The width of the main lobe narrows with the increase of the polynomial degree, and the narrowest level was also observed for the polynomial with the ${\tilde{\Phi }}_{10\;\mathrm{BRM}}$. The obtained result for the polynomial ${\tilde{\Phi }}_{10\;\mathrm{BRM}}$ is, however, sufficient for most radar applications, because the peak to noise ratio is about 50 dB. It can also be seen that for the polynomial ${\tilde{\Phi }}_{8\;\mathrm{BRM}}$ the main lobe of the cross-correlation function is of similar width, but its maximum value is lower by $\Delta_{\max}\chi =6$ dB in comparison to the ${\tilde{\Phi }}_{10\;\mathrm{BRM}}$ approximation. Respectively for the ${\tilde{\Phi }}_{6\;\mathrm{BRM}}$, the main lobe is smaller by $\Delta_{\max}\chi =7.9$ dB and the difference for the ${\tilde{\Phi }}_{4\;\mathrm{BRM}}$ is $\Delta_{\max}\chi =11.4$ dB. It is worth noting that an approximation by the ${\tilde{\Phi }}_{4\;\mathrm{BRM}}$ order polynomial provides an unacceptable main lobe width, disqualifying such reconstruction in practical applications.

**Figure 16:**
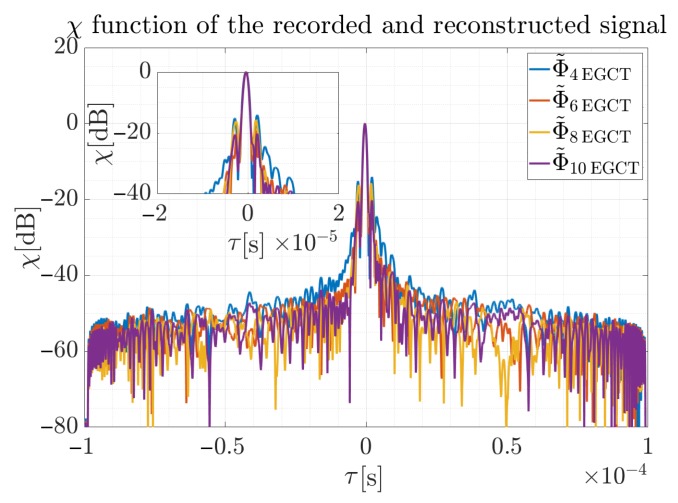
Normalized cross-correlation function of the recorded signal and reconstructed waveform for the analyzed polynomial orders. Reconstructed signal assumed different phase polynomial orders and was provided by the EGCT method.

To verify the results, the EGCT method was used again to reconstruct the signal by four considered polynomials expressed by Equations (21)–(24) using phase models given by Equations (25)–(28). The coefficients of the polynomials are presented in Table 1, and χ function employing the reconstructed and received signal is presented in Figure 16.

**Figure 17:**
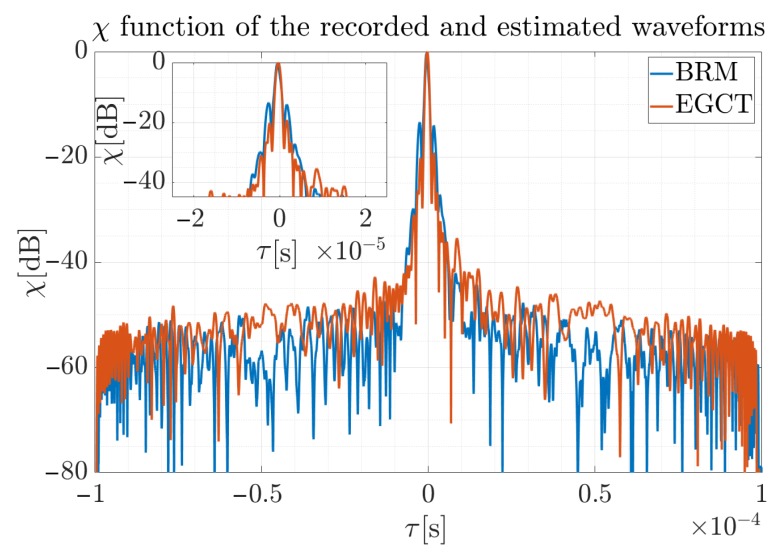
Comparison of the normalized cross-correlation of the recorded signal and reconstructed waveform for two methods considered.

In contrast to the proposed method, the differences between the correlation function for the reconstructed signals using the EGCT method for the various adopted polynomial degrees are smaller. The maximum difference between the peak values of χ is 0.5 dB, which can be neglected. However, BRM is faster and less computationally complex, as shown in [41]. Finally, Figure 17 presents the function of the cross-correlation of the received signal and the reconstructed waveform by two methods. The first one presents χ where the received signal from the ATC radar was correlated with the signal reconstructed by the proposed method, and the second one is the result of the EGCT reconstructed signal (assuming ${\tilde{\Phi }}_{10}$ the phase model for both cases) and the received signal.

The difference in the maximum values for two the methods is $\Delta_{\max}\chi =0.4$ dB and can be omitted in radar applications. It can also be seen that BRM is characterized by slightly higher side lobes for t≈0 s; however, these differences do not exceed 6 dB, while the overall ratio is preserved and the processing was significantly shorter.

In addition, in Table 2 the Peak-to-Side-Lobe-Ratio (PSLR) and peak width were compared for the considered methods, the corresponding parameters for two approaches and the final phase polynomial order were marked bold. Although PSLR for the first side lobe is of a similar level as the one of the first lobe for the LFM cross-correlation function, the remainder of the function is suppressed. This is especially important in the ATC applications for long aircraft-radar ranges. The airplanes in the close vicinity the airports are typically tracked by the airport surveillance radars and precision approach radars which usually have different constructions and are not considered in this paper. In such a case, only distant airplanes located far away from the airports considered, and for such a case the auto-correlation side-lobes are reduced and relatively constant.

Moreover, the comparison presented in Table 3 shows the ratio of multiplication (*C*) for both methods EGCT and CR estimation considering the three estimators described by Equations (15)–(17). The symbols presented in the table are as follows: *N*—FFT size, *M*—signal length, *H*—hop-size, and *L*-number of range profiles calculated for different estimated parameters.

As can be noticed, the CR estimation approach is less computationally complex and requires even hundreds of times fewer multiplications, proving the usability of CR estimation in the TF domain to estimate radar signal parameters in reduced time. During the analysis of the real-life radar pulse, the computation of time measurement was carried out. A comparison was made regarding how long signal parameter estimation takes for the two methods in the case when the polynomial order is known, and for which polynomial order the results are most accurate. The calculations were obtained using a MATLAB 2018b installed on a computer using an Intel i7-7700HQ 2.8 GHz processor, 16 GB DDR4 RAM, an SSD hard drive and a 64-bit Windows 10 system. The calculation of the parameters in the proposed method were assumed as follows: N=4096, M=3950 Sa, H=1, k=1000 and for the EGCT analysis N=4096, L=1001. The results are presented in Table 4.

The proposed method based on the CR estimation in the TF domain is significantly faster, and the difference increases for a higher number of polynomials considered. The algorithm can be used in real systems as a preliminary signal reconstruction method. In this case, the first reconstruction is carried out using the proposed method for all polynomials taking into account small discrepancies; then, the most accurate is chosen among them. For this purpose, for example, the Pearson correlation coefficient can be used to provide information about which polynomial order is the most effective. Then, the precise reconstruction can be done, which in reference to Table 4 reduces the time from 1450 [s] to 89[s]+584[s]=673[s]. The implementation time in the MATLAB environment was taken into consideration, and when using GPUs and dedicated architecture, this time could be significantly reduced.

## 7. Conclusions

In this paper, a new approach to NLFM pulse reconstruction has been presented which can be applied in ELINT, ER, EW systems and passive bistatic radars using other radars as a source of illumination. The results obtained for the proposed method have been compared with the known and accurate EGCT method based on the MLE, allowing precise signal reconstruction to be carried out. However, the mentioned algorithm is computationally complex, and in cases when the parameters of the transmitter are unknown, accurate phase coefficients estimation is time-consuming. Such a problem was precisely described in [41] and the previous section, where several mathematical operations were compared for both the EGCT method and the CR estimation approach. From the signal recognition point of view, the cross-correlation function is enough to compare reconstruction quality, so such a comparison was performed to confirm that the proposed algorithm provides negligibly small disparity when compared to a matched-filtration-based method (EGCT). An additional advantage of the introduced approach is the possibility to use it in other fields, such as hydrolocation. The only difference can be the approximating function, which can be a polynomial of another type, or a different function entirely. The main disadvantage of the BRM algorithm is very poor reconstruction precision for the mismatched approximating polynomial order. However, as mentioned in the previous section this method can be used as a preliminary reconstruction providing initial information about the signal parameter. In the future, the authors want to apply the proposed approach in a real radar system employing a CUDA platform and/or FPGA circuit, allowing real-time reconstruction to be performed. Additionally, a different type of approximation can be used, such as RANSAC (RANdom SAmple Consensus), to speed-up and enhance the algorithm. The last concept in particular seems to be interesting due to the ability to approximate the function in the presence of high noise, and many outliers which can be useful in the case when the reference signal has a relatively low SNR. An additional concept is to apply adaptive STFT analysis, employing a window width dependent on the signal parameters, e.g., CR, which can affect the improvement of the TF energy distribution for fast changing signals.

## Figures and Tables

**Table 1:** Estimated phase coefficients for different polynomials.

${\tilde{\Phi }}_{x\;\mathrm{EGCT}}$	Linear FM Component	Non-Linear FM Component
x=4	$\tilde{\alpha }=-7.4761\times 10^{9}$	$\tilde{\beta }=-2.0500\times 10^{18}$
x=6	$\tilde{\alpha }=-8.1233\times 10^{9}$	$\tilde{\gamma }=-1.1483\times 10^{27}$
x=8	$\tilde{\alpha }=-8.4352\times 10^{9}$	$\tilde{\delta }=-6.2643\times 10^{35}$
x=10	$\tilde{\alpha }=-8.6326\times 10^{9}$	$\tilde{\varepsilon }=-3.2627\times 10^{44}$

**Table 2:** A comparison of PSLRs and the peak widths for the considered methods.

Estimated Chirp Phase ${\tilde{\Phi }}_{x}$	PSLR EGCT [dB]	PSLR BRM [dB]	Peak Width EGCT [μs]	Peak Width BRM [μs]
x=4	−14.09	−0.1	0.99	4.4
x=6	−18.97	−0.35	0.93	3.8
x=8	−15.79	−0.26	0.85	4.2
x=10	−20.24	−14.28	**0.86**	**0.93**

**Table 3:** Number of multiplication operations for the analyzed methods.

Case	Parameters	$C_{\mathrm{EGCT}}/C_{K}$	$C_{\mathrm{EGCT}}/C_{D,F}$
I	M=4000 N=1024 H=10 L=100	0.32	0.18
II	M=4000 N=1024 H=10 L=1000	3.16	1.83
III	M=4000 N=1024 H=100 L=1000	31.57	18.27
IV	M=4000 N=1024 H=100 L=10,000	315.66	182.75

**Table 4:** Algorithms execution time.

Method	One Polynomial	Four Polynomials
EGCT	584 [s]	1450 [s]
BRM	87 [s]	89 [s]
